# An evaluation of a national oral rehydration solution and zinc scale-up program in Kenya between 2011 and 2016

**DOI:** 10.7189/jogh.09.010505

**Published:** 2019-06

**Authors:** Felix Lam, Leslie Wentworth, Peter Cherutich, Santau Migiro, Khadija Abdala, Michael Musyoka, Samuel Ogolla, McDonald Obudho, Zachary Mwangi, Rosemary Kihoto, Collins Cheruiyot, Betty Wariari, Audrey Battu, Kate Schroder

**Affiliations:** 1 Clinton Health Access Initiative, Boston, Massachusetts, USA; 2 Department of Preventive and Promotive Health, Ministry of Public Health and Sanitation, Nairobi, Kenya; 3 Newborn, Child, and Adolescent Health Unit, Ministry of Public Health and Sanitation, Nairobi, Kenya; 4 Kenya National Bureau of Statistics, Nairobi, Kenya; 5 Clinton Health Access Initiative, Nairobi, Kenya

## Abstract

**Background:**

In Kenya, diarrheal disease is the second leading cause of death among children under five. The Government of Kenya launched a national plan to increase coverage of oral rehydration solution (ORS) and zinc by addressing demand and supply-side barriers. This study evaluates progress of ORS and zinc uptake in Kenya according to the national plan from 2011 to 2016.

**Methods:**

In 2016, we conducted a nationally representative population-based household survey to estimate coverage of ORS and zinc for treatment of diarrhea cases among children under five. We also used ORS and zinc coverage data from the two most recent Kenya Demographic and Health Surveys in 2008/09 and 2014 to estimate annual changes in coverage rates during the program period. Based on these inputs, we used the Lives Saved Tool to estimate the number of diarrhea deaths averted between 2011 and 2016 due to increased use of ORS and zinc.

**Results:**

The 2016 survey results showed that ORS coverage was 42% (95% confidence interval (CI) = 38%, 47%) and zinc coverage was 18% (95% CI = 15%, 23%). The estimated coverage for the combined use of ORS and zinc was 15% in 2016 (95% CI = 12%, 19%). Compared to 2011, an additional 3340 (sensitivity bounds = 2 670, 3 920) diarrhea deaths among children under five were averted due to increases in ORS and zinc coverage.

**Conclusions:**

Kenya was successful in catalyzing uptake of combined treatment with ORS and zinc, which rose from 0.8% in 2011 to 15% in 2016. Ongoing efforts are necessary to further build on these gains.

## Background

Diarrheal disease is the second leading cause of death among children under five years in Kenya [1]. In 2011, there were approximately 19 million episodes of diarrhea and an estimated 9500 deaths in children under five due to diarrhea [2]. Oral rehydration solution (ORS) and zinc are recommended by the World Health Organization (WHO) and United Nations Children’s Fund (UNICEF) to treat diarrhea in children [3]. ORS can prevent up to 93% of diarrhea deaths in children and zinc has been shown to reduce the duration, severity, and reoccurrence of diarrhea [4,5]. Both treatments cost less than $0.50. The Government of Kenya (GOK) adopted ORS into the national guidelines for pediatric diarrhea in the 1980s and updated the guidelines to include zinc in 2007. [6] However, the 2008-09 Kenya Demographic and Health Survey (KDHS) found that only 39% of children received ORS and only 0.2% received zinc for treatment of diarrhea [7]. The low coverages were driven by several key barriers, including low knowledge of recommended treatment by caregivers and providers, limited availability of zinc and ORS, expensive products in private outlets, and policies restricting zinc to patients with a prescription. To accelerate progress, the Ministry of Public Health and Sanitation (MOPHS), with the support from the Clinton Health Access Initiative (CHAI) and with participation of implementing partners such as the United Nations Children’s Fund (UNICEF), PATH, and Micronutrient Initiative (MI), launched a 5-year national plan to increase uptake of ORS and zinc to 90% between 2011 and 2016 [6].

Since the start of the program implementation, results from the KDHS conducted in 2014 found that ORS and zinc coverage had increased to 8% (from less than 1% in 2008-09) [8]. In this study, we aim to update coverage estimates for 2016 in order to evaluate changes in ORS and zinc coverage during the program period.

## Study objective

The primary objective of this study is to measure ORS and zinc coverage in 2016 and to evaluate coverage changes since 2011. Second, the study aims to estimate the number of diarrheal deaths averted due to coverage changes between 2011 and 2016.

## Methods

### Program description

Kenya’s efforts to improve pediatric diarrhea treatment rates focused on four key domains: (1) developing supportive policy, which included allowing zinc to be dispensed over-the-counter and shifting government procurement to co-packaged ORS and zinc; (2) creating a sustainable and competitive market for ORS and zinc to improve availability and affordability; (3) increasing consumer demand for ORS and zinc; and (4) improving health provider practices in the management of pediatric diarrhea. **Table 1** summarizes the activities by each domain.

**Table 1:** Key activities implemented

Intervention area	Activities
Policy	OTC status for zinc
	National scale-up plan to align and optimize efforts across partners
	Co-pack procurement in public sector
Supply	Co-pack supplier engagement
	New brands registered
Caregivers/Community	Mass media
	Clinic-based health talks
	ORT corners
Healthcare providers	IMCI trainings and CMEs
	Supportive supervisions
	IMCI app
	One-on-one educational visits to private providers

OTC – over-the-counter; ORT – oral rehydration therapy; IMCI – integrated community case management; CME – continuing medical education

One of the steps to developing a supportive policy environment was to increase access to zinc in private retail outlets by allowing zinc to be dispensed over-the-counter and enabling sales alongside ORS. The policy change also allowed for zinc to be marketed directly to caregivers and patients, which is prohibited for prescription medicines. This policy change was achieved in 2012. Second, to ensure ORS and zinc were administered together, the GOK recommended adoption of co-packaged ORS and zinc to all health facilities. Starting in 2015, the Kenya Medical Supplies Authority (KEMSA), a state corporation which oversees procurement and distribution of medical supplies, switched their procurement of pediatric diarrhea treatments from individually packaged units of ORS and zinc to co-packaged ORS and zinc. KEMSA also updated their product specifications to improve ease of administration and meet patient preferences. KEMSA’s new specifications required that zinc tablets were dispersible and taste-masked and that ORS was flavored. These technical updates [9,10], in addition to the revision of the national policy on management of diarrhea to include zinc, were broadly disseminated.

**Figure 1:**
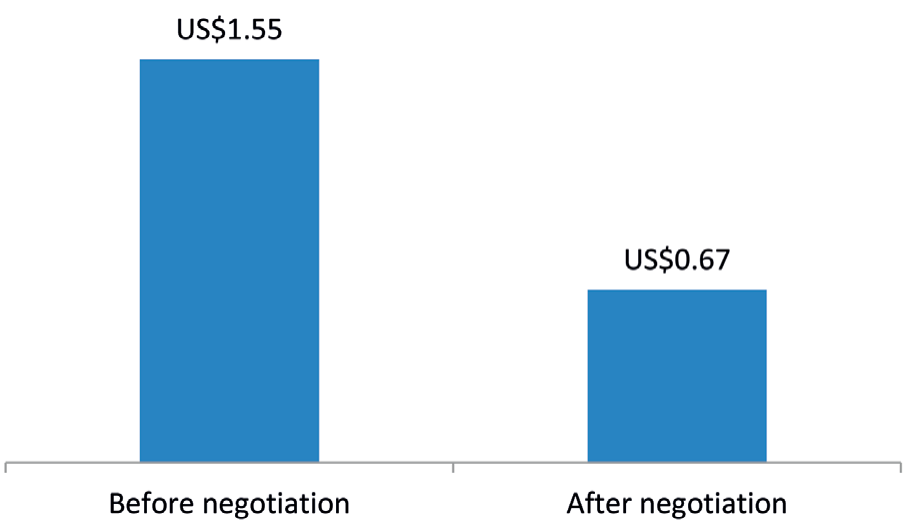
Retail price of full treatment course* before and after price negotiation. *Full treatment course = four 500mL oral rehydration solution sachets and 10 zinc tablets

**Figure 2:**
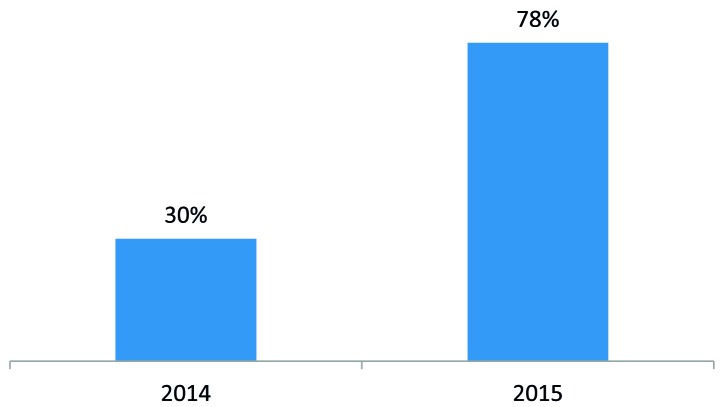
Percent of private pharmacies with both ORS and zinc in-stock on the day of the visit in 2014 and 2015. ORS – oral rehydration solution

To develop a self-sustaining and competitive market for ORS and zinc, the program engaged with three local manufacturers of ORS and zinc – Cosmos, Universal, and Laboratory & Allied – to introduce co-packaged ORS and zinc starting in 2015 and to negotiate affordable prices. The development of a co-pack and price negotiations led to a reduction in the retail price of a full treatment course (four 500mL ORS sachets and 10 zinc dispersible tablets) from US$1.55 to US$0.67 (**Figure 1**). In addition, the program worked with marketing agencies to develop promotional materials on ORS and zinc and freely provided the promotional materials to suppliers for distribution by their sales teams during routine visits to private medicine outlets. Program monitoring data showed that the percent of private medicine outlets stocking both ORS and zinc increased from 30% in 2014 to 78% in 2015 (**Figure 2**).

To increase demand for ORS and zinc among caregivers, the program launched radio spots in the local language promoting ORS and zinc. The spots were designed to improve diarrhea prevention and treatment practices and to encourage prompt care seeking. To reinforce the mass media messages, interpersonal outreach was conducted through clinic-based “health talks” that targeted women attending routine antenatal care (ANC), immunization and well-baby visits. Standardized, illustrated “health talk kits” that included a pictorial chart booklet covering key child health topics, such as diarrhea management, vaccines, nutrition, and breastfeeding, were developed and provided to health workers. Health workers used these communication materials to provide daily informational sessions to large groups of caregivers who were waiting for appointments.

The MOPHS also re-introduced oral rehydration therapy (ORT) corners within health facilities. ORT corners aim to provide on-site rehydration for sick children and, at the same time, give caregivers hands-on experience to learn how to correctly prepare and administer ORS to their sick children. It has been a successful strategy in previous programs throughout the world [11,12].

Lastly, to build provider capacity, the MOPHS expanded trainings on Integrated Management of Childhood Illnesses (IMCI) through platforms such as didactic trainings, continuous medical education (CME) programs, mentorship and supportive supervisions, and on-the-job trainings. After updating the diarrhea management policy to include zinc, the MOPHS conducted refresher trainings across the country. The program streamlined IMCI trainings to one week of clinical and didactic trainings, with a cascading peer-to-peer model where trained clinicians were responsible for training all other clinicians who treat pediatric patients at their facility. IMCI certification was dependent on an assessment of the facility’s compliance, not just the trained clinician’s compliance, with the IMCI protocol six weeks after the training. The model also included routine supportive supervision by county and sub-county leadership to confirm ongoing compliance and help address any gaps, particularly challenges related to staff turnover. The program also created an IMCI mobile app to make the guidelines readily available to anyone using a smartphone.

### Evaluation design and setting

The 2016 study was designed as a cross-sectional household survey to measure coverage of ORS and zinc. We also used Spectrum v5.753 Lives Saved Tool (LiST) (Avenir Health, Glastonbury, CT USA), with ORS and zinc coverage results from existing national household surveys and this study’s cross-sectional household survey to model the estimated number of diarrheal deaths averted due to changes in coverage between 2011 and 2016. LiST is a deterministic mathematical modelling software that uses intervention coverage inputs and literature on the effect of those interventions on cause-specific mortality reduction in order to attribute mortality reductions due to changes in intervention coverage. LiST has been used to help guide policymakers in setting priorities for investments in interventions and to evaluate the impact of programs aiming to scale up proven interventions. The theoretical approach, modeling structure, and specific methods for LiST are published elsewhere [13,14].

### Survey study population

The study population was children age 0-59 months who had diarrhea in the two weeks preceding the survey.

### Survey sampling design

The survey sample was designed to be nationally representative. The sample was drawn from the Fifth National Sample Survey and Evaluation Programme (NASSEP V). This is a frame that the Kenya National Bureau of Statistics (KNBS) uses to conduct household-based surveys throughout Kenya. The NASSEP V contains 96 251 clusters and is stratified into 47 counties, and within each county, further stratified into urban and rural strata. KNBS used two-stage cluster randomized sampling to select households for inclusion in the study. In the first stage, clusters were stratified by county and urban/rural areas and KNBS randomly selected 560 clusters (279 urban clusters and 281 rural clusters) with probability proportional to size. In the second stage, 25 households were randomly selected from each cluster using the latest household listing available for the cluster.

### Survey data collection procedures

KNBS hired data collection personnel and took them through five days of training. The data collection personnel were selected based on their experience in conducting previous household-based surveys. Seventy enumerators and 24 supervisors were engaged to implement the study. The training covered the sampling approach, survey tools, use of the electronic data collection platform SurveyCTO (Dobility Inc, Boston MA USA), research ethics, and practice in the field. The data collection commenced on November 2, 2016 and was completed on December 7, 2016.

The survey data collection tool was adapted from the KDHS, specifically the household members listing section and the child illness section. The enumerators listed all the de facto members in the household including their ages. They then asked whether any of the children under five had diarrhea in the two weeks preceding the survey. If the child did have diarrhea in the last two weeks, the enumerator asked questions regarding the care and treatment for the child, including where care was sought and what treatments were given. The tool was piloted and revised based on feedback from the field data collection personnel. A team of three supervisors and nine enumerators were engaged in the pilot. In addition, to aid in memory recall, picture cards of local ORS, zinc, antibiotics, anti-diarrheal medicines, and traditional medicines were developed, tested, and deployed during fieldwork.

Upon reaching the selected clusters, data collection teams met with the area village elders to explain the purpose of the study and seek their support in locating the selected households. Once the selected households were identified, data collectors requested to speak with the main caretaker of children in the household, explained the purpose of the study, requested written informed consent from the respondent, and if consent was given, conducted the interview.

### Data analyses

Descriptive analyses were conducted on three primary outcomes:

1. The proportion of children under five with diarrhea in the last two weeks that received ORS
2. The proportion of children under five with diarrhea in the last two weeks that received zinc
3. The proportion of children under five with diarrhea in the last two weeks that received both ORS and zinc

The outcomes were disaggregated by socioeconomic status (SES) and location of the household (urban or rural), and we conducted two-sample *t* tests to examine whether the outcomes differed by SES or location. SES was represented by wealth quintiles. To construct the wealth quintiles, principle component analysis using household assets were conducted [15]. The urban and rural definitions provided by the NASSEP V sampling frame were used to categorize households.

Sampling design weights and non-response weights were calculated based on the NASSEP V sampling frame and incorporated into all analyses. The analysis was conducted using Stata 14 (StataCorp, College Station TX, USA).

### LiST statistical analyses

The study used KDHS 2003, KDHS 2008-09, KDHS 2014, and the 2016 survey described above as direct inputs into the LiST model for ORS and zinc coverage in 2003, 2008, 2014, and 2016, respectively. For 2011, the year the program initiatives began, ORS coverage was estimated by linearly projecting forward the average annual coverage rate increases between 2003 and 2008. Zinc was not included in the 2003 KDHS and was not part of the national guidelines until 2007, therefore, we assumed zinc coverage was 0% in 2007. We used the rate of increase between 2007 and 2008 to estimate zinc coverage for 2011. We estimated ORS and zinc coverage for all years without direct survey data (eg, 2012, 2013, and 2015) by linearly projecting forward the average annual coverage rate increases between survey periods.

We also estimated coverages of other interventions included in the LiST software (eg, prevalence of exclusive breastfeeding, vaccine coverage, etc.) using the KDHS 2008-09 and KDHS 2014 results. As above, we used linear interpolation to estimate coverage of these additional interventions for all years in between 2008 and 2014. We did not gather coverage of other interventions in the 2016 study’s survey. To obtain 2016 coverage estimates, we projected forward the annual linear change in coverage from 2008 to 2014 to estimate the coverage for 2015 and 2016. The aim of projecting forward coverage estimate trends to 2016 was to produce conservative estimates of the diarrhea deaths averted attributable to ORS and zinc coverage changes. The LiST model first attributes deaths averted to preventative and protective interventions (eg, exclusive breastfeeding, vaccines, etc.) and the remaining deaths averted are then attributed to treatment interventions (eg, ORS and zinc).

Using LiST, we calculated the number of additional diarrheal deaths averted attributable to each child health intervention, compared to a counterfactual in which coverage of all interventions were kept constant at 2011 values. We also calculated lower and upper sensitivity bounds, which are the number of diarrheal deaths averted using the lowest and estimates of intervention effectiveness used by LiST.

### Ethical approval

Ethical approval for the study was received from Kenya Medical Research Institute (Protocol #: KEMRI/RES/7/3/1).

## RESULTS

### Survey results

**Figure 3:**
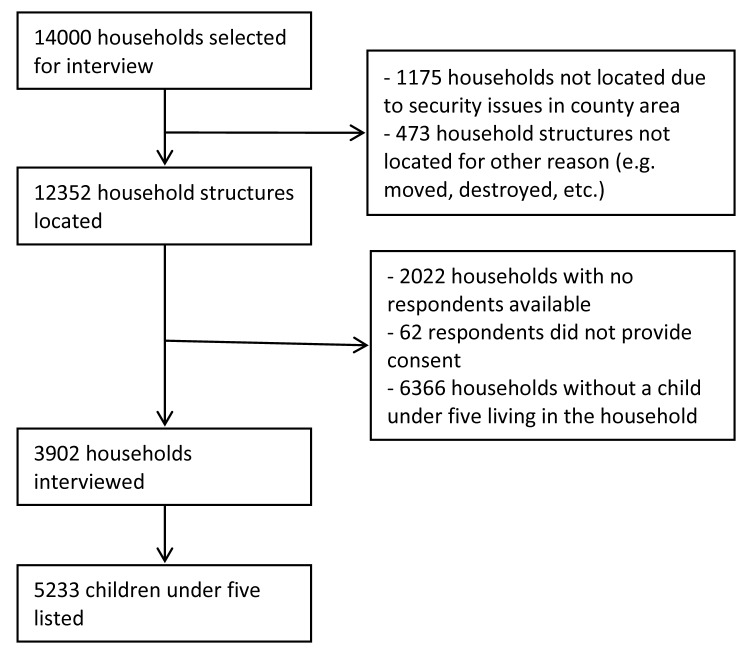
Sampling results.

**Figure 3** presents the results of the sampling and listing exercise. Of the 14 000 households sampled from the NASSEP V sampling frame, we were able to find the household structures for 12 352 households. Of that, 2022 respondents were unavailable at the time of the visit, 62 respondents declined to participate, and 6366 did not have a child under five living in the household. We completed interviews with 3902 households and found 5233 children living in those households.

Of the children under five, we found 14% (95% confidence intervals (CI) = 13%, 15%) had diarrhea in the last two weeks. **Table 2** presents outcomes and selected characteristics of children who had diarrhea in the last two weeks. Among children with diarrhea, we found 42% (95% CI = 38%, 47%) received ORS, 18% (95% CI = 15%, 23%) received zinc, and 15% (95% CI = 12%, 19%) received both ORS and zinc. Most caregivers of the children sought advice or care for the diarrhea case outside the home – 63% (95% CI = 58%, 68%). Of those that did seek care, 70% (95% CI = 64%, 76%) sought care from a public health care source and 23% (95% CI = 18%, 29%) sought care from a private health care source. A small proportion – 4% (95% CI = 2%, 7%) – sought care from a non-medical source (eg, friend, family, or traditional healer), and only 3% (95% CI = 1%, 7%) sought care from multiple sources.

**Table 2:** Selected outcomes and characteristics of children with diarrhea in the last two weeks (N = 721)

	Proportion (95% CI)
**Outcomes:**	
Received ORS	42% (38%, 47%)
Received zinc	18% (15%, 23%)
Received both ORS and zinc	15% (12%, 19%)
Sought care or advice outside the home	63% (58%, 68%)
**Selected child characteristics:**	
Rural	64% (58%, 70%)
Female	45% (41%, 50%)
0-11 mo	23% (19%, 27%)
12-23 mo	27% (23%, 31%)
24-35 mo	23% (19%, 27%)
36-47 mo	16% (14%, 20%)
48-59 mo	11% (9%, 15%)
Received rotavirus vaccination	50% (45%, 54%)
**Selected illness characteristics:**	
Mean duration of diarrhea	3.9 (3.7, 4.2)
Bloody stool	11% (9%, 14%)
No other accompanying symptoms	37% (33%, 41%)
Fever	36% (32%, 41%)
Vomiting	13% (11%, 16%)
Cough	12% (9%, 15%)
Unable to eat	11% (9%, 15%)
Restless or easily irritable	8% (6%, 12%)
Lethargy, fatigue, or no energy	7% (5%, 10%)
**Source of care:**	
Sought care from public health care sources	70% (64%, 76%)
Sought care from private health care sources	23% (18%, 29%)
Sought care from other non-medical sources (friend or family)	4% (2%, 7%)
Sought care from multiple sources	3% (1%, 7%)

ORS – oral rehydration solution

**Table 3** presents ORS and combined ORS and zinc coverage by wealth quintile and urban/rural area. ORS coverage was 43% (95% CI = 37%, 49%) in rural areas and 41% (95% CI = 35%, 47%) in urban areas. For the poorest and wealthiest quintiles, ORS coverage was 48% (95% CI = 40%, 57%) and 31% (95% CI = 21%, 42%), respectively. Combined ORS and zinc coverage was 15% (95% CI = 11%, 21%) in rural areas and 15% (95% CI = 10%, 21%) in urban areas. Among the poorest and wealthiest quintiles, combined ORS and zinc coverage was 21% (95% CI = 14%, 33%) and 14% (95% CI = 7%, 26%), respectively. We did not find a statistically significant difference in the odds of using ORS (*P* = 0.61) or combined ORS and zinc (*P* = 0.93) by urban or rural locations. We did find the odds of using ORS was 0.47 (95% CI = 0.25, 0.87) times lower among the wealthiest quintile compared to the poorest quintile (*P* = 0.02). We did not find any statistically significant difference in the odds of using combined ORS and zinc by wealth quintile (*P* = 0.28).

**Table 3:** ORS and zinc coverage by urban/rural and wealth

	ORS use			Combined ORS and zinc use		
**Attribute**	**Proportion (95% CI)**	**OR (95% CI)**	***P*-value**	**Proportion**	**OR**	***P*-value**
**Wealth:**						
Poorest quintile	48% (40%, 57%)	Ref		22% (14%, 33%)	Ref	
Wealthiest quintile	31% (21%, 42%)	0.47 (0.25, 0.87)	0.02	14% (7%, 26%)	0.58 (0.21, 1.57)	0.28
**Location:**						
Rural	43% (37%, 49%)	Ref		15% (11%, 21%)	Ref	
Urban	41% (35%, 47%)	0.91 (0.63, 1.31)	0.61	15% (10%, 21%)	0.98 (0.56, 1.71)	0.93

CI – confidence interval, OR – odds ratio, ORS – oral rehydration solution

### LiST model results

Table S1 in **Online Supplementary Document** presents the estimated coverages for child health interventions used in the LiST model. We estimated that in 2011, 45% of children with diarrhea received ORS and 0.8% received zinc. Using these estimates, the 2014 KDHS, and our own survey as inputs for the LiST model, we estimated that, compared to the counterfactual, an additional 3340 (sensitivity bounds: 2670, 3920) diarrheal deaths were averted between 2011 and 2016 due to changes in ORS and zinc coverage. The number of diarrheal deaths averted was greatest in 2014 when ORS coverage reached 54% and zinc coverage was 8%, and prior to the full implementation of devolution (see Discussion). Compared to if coverage remained at 2011 values, we estimated an additional 1330 (sensitivity bounds: 1090, 1520) diarrheal deaths were averted in 2014.

Rotavirus vaccination, which was introduced in Kenya in 2014, was estimated to avert 1190 (sensitivity bounds: 550, 1590) additional diarrheal deaths. The estimated coverage of rotavirus vaccine was 0% in 2011 and 74% in 2016. Age-appropriate breastfeeding was estimated to avert an additional 730 (sensitivity bounds: 300, 1010) diarrheal deaths with coverage of exclusive breastfeeding for children less than 1 month having increased from 68% in 2011 to 76% by 2016.

## DISCUSSION

Kenya has made progress in reducing child mortality due to diarrhea. Coverage of the optimal treatment – combined ORS and zinc – has reached 15% in 2016 from 0.8% in 2011, and gains were equally strong in rural and poor households, who are more vulnerable and more likely to be at risk of mortality. We also estimated the increases in ORS and zinc coverage averted 3340 deaths between 2011 and 2016 and preventative and protective interventions such as rotavirus vaccination and breastfeeding also contributed substantially to reducing mortality due to diarrhea. If coverage increases are maintained or continue to rise, many more deaths will be averted.

Despite these accomplishments, coverage remains relatively low. One of the major challenges faced was the devolution of the government. In 2010, Kenya adopted a constitution that decentralized government functions from the national legislature and executive branch to each of the 47 counties. One of the functions that became decentralized was the purchasing of medicines, which had previously been centrally managed by KEMSA. Between 2013 and 2014, county governments took on the responsibility for budgeting and ordering medicines. This fundamental change in procurement for all essential medicines created challenges with making ORS and zinc available as counties adjusted to this entirely new, highly complex responsibility. This broader change in Kenya’s procurement system was a strong headwind to the efforts by MOPHS and partners to increase ORS and zinc usage, and gains may have been greater in the absence of this. Going forward, it will be important to continue to strengthen procurement at the county level to ensure consistent availability of all essential medicines, including ORS and zinc.

High turnover rates of trained health staff was also a challenge. A six-month follow up to facilities trained in IMCI found that 20% of the trained staff had left their post. High turnover impeded the ability of facilities to maintain high levels of compliance with IMCI guidelines. This finding underscores the importance of using ongoing mentorship and supportive supervision to encourage compliance with recommended IMCI practices and updated guidelines.

The challenges and lessons from this program may provide valuable insights for other governments and partners seeking to increase coverage of ORS and zinc. Overall, the comprehensive approach to address the four domains likely contributed to increased coverage of ORS and zinc. A comprehensive program addressing policy, supply, and demand barriers have been proven successful in other contexts as well, such as Bangladesh, India, Nepal, Nigeria, and Uganda. [16-21] In particular, introduction of co-packaged ORS and zinc in public sector facilities likely accelerated uptake of the combined treatment. The combination of ORS and zinc is the optimal treatment for diarrhea, and co-packaging is a novel approach to increase the likelihood that both treatments are offered together. A cluster randomized trial in Ethiopia found that co-packaging ORS and zinc significantly increased adherence [22]. In addition, efforts to bring new suppliers of ORS and zinc into the market – including domestic manufacturers – helped to increase competition and facilitate more competitive pricing. The strong leadership of the Kenyan government at both national and county levels also contributed to the pace and quality of scale up, especially through strong policy guidance, direct investment in consumer demand generation, and the involvement of county health management teams in routinely monitoring IMCI adherence.

A limitation of this study is that a household survey was not conducted in 2011 when the efforts first began. To address this limitation, we used the KDHS 2003 and 2007-08 surveys to estimate coverage in 2011. Another limitation of the study was that we did not update the household listing in the selected clusters prior to sampling. This likely led to the high rates of households that could not be found or contacted for interviews and further limited the power of the study to perform statistical tests. Limited funding prevented the study from updating the household listings in selected clusters.

While the LiST methodology relies on the best available research to inform its mathematical model and has been validated, the LiST model relies on accurate inputs. The household survey we conducted was limited in scope to diarrhea management, and therefore, we did not collect coverage information on other interventions. Preventative interventions, such as handwashing and measles and rotavirus vaccination, reduce the overall mortality pool and thus the number of diarrheal deaths that can be averted by ORS and zinc. We try to address this by using the KDHS 2007-08 and 2014 to estimate coverage of those interventions in 2016.

The study design was also not conducive to conduct inferential analysis in order to isolate or relate ORS and zinc coverage changes to specific program activities. Future evaluations should consider evaluation designs that would allow for testing the impact of program interventions, such as a stepped-wedge trial design. Though the program was a national scale-up effort, interventions could have been phased to different geographies over time so that there would have been control areas against which to test the effect of program interventions before introducing the interventions to the control areas.

Lastly, several of the study investigators were also involved in the program implementation. We acknowledge that there may be inherent bias to publish positive results in this study; however, the study survey was implemented by KNBS, a national institution in charge of producing rigorous statistical data for Kenya. KNBS was not involved in any the program design or implementation. Coverage results used in the LiST model also relied on the DHS surveys, which were independently collected using standardized methods.

## CONCLUSION

Kenya has been successful in catalyzing uptake of combined treatment with ORS and zinc from 0.8% in 2011 to 15% in 2016, and ongoing efforts are needed to further build on these gains.

## Additional material

Online Supplementary Document
