# Supplementary material for: An evaluation of a national oral rehydration solution and zinc scale-up program in Kenya between 2011 and 2016
Source: J Glob Health. 2019 Jun 29;9(1):010505. doi: 10.7189/jogh.09.010505 (PMC6608604; doi:10.7189/jogh.09.010505)
Supplement: Online Supplementary Document [file jogh-09-010505-s001.pdf]

J Glob Health 2019; 9: 010505

Online Supplementary Document

Lam et al. An evaluation of a national ORS and zinc scale-up program in Kenya between 2011 and 2016

Contents

Table S1. LiST inputs and sources for child health interventions 2

Table S1. LiST inputs and sources for child health interventions

Intervention	2011	2012	2013	2014	2015	2016	Sources
ORS - oral rehydration solution	44.6	47.6	50.7	53.8	48.0	42.2	KDHS 2008; KDHS 2014; KNBS/CHAI 2016
Zinc for treatment of diarrhea	0.3	2.9	5.5	8.1	13.2	18.3	KDHS 2008; KDHS 2014; KNBS/CHAI 2016
Skilled birth attendance	54.6	57.9	61.2	64.5	67.8	71.1	KDHS 2008; KDHS 2014
Health facility delivery	52.0	55.2	58.3	61.5	64.6	67.7	KDHS 2008; KDHS 2014
Exclusive breastfeeding for <1 month	68.0	68.7	69.4	70.2	73.2	76.3	KDHS 2008; KDHS 2014
Prevalence of early initiation of breastfeeding	59.0	59.7	60.4	61.1	62.0	62.8	KDHS 2008; KDHS 2014
Exclusive breastfeeding for 1-5 month	44.2	45.5	46.8	48.1	51.2	54.3	KDHS 2008; KDHS 2014
Any breastfeeding 6-11 month	97.2	97.2	97.2	97.2	97.3	97.5	KDHS 2008; KDHS 2014
Any breastfeeding 12-23 month	73.6	73.6	73.6	73.6	73.9	74.2	KDHS 2008; KDHS 2014
Complementary feeding	39.8	40.2	40.5	40.9	41.3	41.6	KDHS 2008; KDHS 2014
Vitamin A supplementation	37.0	66.0	19.0	28.0	37.0	41.0	UNICEF
Improved sanitation - Utilization of latrines or toilets	29.4	29.6	29.9	30.1	30.1	30.3	WHO-UNICEF washdata.org
Improved water source	60.8	61.6	62.3	63.1	63.2	63.9	WHO-UNICEF washdata.org
Water connection in the home	21.1	21.3	21.4	21.6	21.7	21.8	WHO-UNICEF washdata.org
Hand washing with soap	13.7	13.7	13.8	13.9	14.0	14.1	WHO-UNICEF washdata.org
Hygienic disposal of children's stools	80.7	81.5	82.3	83.1	83.9	84.7	KDHS 2008; KDHS 2014
BCG - Single dose	99.0	97.0	92.0	94.0	87.0	99.0	WHO-UNICEF
Polio - Three doses	97.0	94.0	94.0	93.0	83.0	88.0	WHO-UNICEF
Pentavalent	96.0	94.0	87.0	92.0	89.0	89.0	WHO-UNICEF
Pneumococcal - Three doses	85.0	82.0	75.0	81.0	75.0	78.0	WHO-UNICEF
Rotavirus - Two doses	0.0	0.0	0.0	19.0	66.0	74.0	WHO-UNICEF
Meningococcal A - Single dose	0.0	0.0	0.0	0.0	0.0	0.0	WHO-UNICEF
Measles - Single dose	87.0	93.0	94.0	95.0	96.0	96.0	WHO-UNICEF
Oral antibiotics for pneumonia	60.8	62.5	64.1	65.7	67.4	69.0	KDHS 2008; KDHS 2014
ACTs for treatment of malaria	11.2	11.9	12.5	13.1	15.0	15.0	KDHS 2008; KDHS 2014
